# Effect of EGFR-TP53 co-mutation on the efficacy of EGFR-TKIs in patients with advanced NSCLC and therapeutic strategies: A retrospective study

**DOI:** 10.1097/MD.0000000000049446

**Published:** 2026-06-19

**Authors:** Xiaohan Feng, Lei Liu

**Affiliations:** a The Respiratory Department, China Medical University, The General Hospital of Northern Theater Command Training Base for Graduate, Shenyang, Liaoning, China; b The Respiratory Department, General Hospital of Northern Theater Command, Shenyang, Liaoning, China.

**Keywords:** co-mutation, EGFR, non-small cell lung cancer, TP53, tyrosine kinase inhibitors

## Abstract

This study aimed to describe the therapeutic efficacy and clinical outcomes of patients with advanced non-small cell lung cancer harboring both EGFR mutation and TP53 co-mutations compared to those with only EGFR mutation (TP53 wild-type) when treated with EGFR-tyrosine kinase inhibitors (EGFR-TKIs) and explore strategies to optimize treatment approaches for patients with EGFR-TP53 co-mutation. Demographic information and clinical data of target population were collected for analysis. Forty-six patients who met the specified criteria included 20 patients with EGFR mutation and TP53 wild-type and 26 patients with EGFR-TP53 co-mutation. The objective response rate of the EGFR-TP53 co-mutant group and EGFR mutation TP53 wild-type group was 46.2% versus 75.0% (*P* = .049); median PFS (mPFS) was 8.0 months vs 18.0 months (*P* = .009); and mOS was 11.0 months versus 31.0 months (*P* = .021). The mPFS of the EGFR-TP53 co-mutants receiving gefitinib and osimertinib as first-line therapy was 3.0 months versus 9.0 months (*P* = .022) and mOS was 6.0 months versus 12.0 months (*P* = .041). The mOS of the EGFR-TP53 co-mutant group who received second-line TKIs combined with platinum-containing double-drug chemotherapy and bevacizumab after the progression of first-line single-drug TKIs was 27.0 months versus 6.0 months compared with those who did not receive second-line therapy (*P* = .019). TP53 mutations can significantly negatively affect the efficacy of EGFR-TKIs in patients with advanced non-small cell lung cancer with EGFR mutation. In first-line EGFR-TKIs monotherapy in patients with EGFR-TP53 co-mutation, osimertinib was clearly superior to gefitinib. In first-line EGFR-TKIs monotherapy progression, TKIs combined with chemotherapy and antiangiogenesis therapy could prolong patients’ survival.

## 1. Introduction

Non-small cell lung cancer (NSCLC) is the leading cause of cancer-related deaths worldwide, causing nearly 1.8 million deaths each year.^[1]^ At present, there are many targeted drugs available clinically. Epidermal growth factor receptor (EGFR) mutation is the most common driver gene mutation in NSCLC patients,^[2]^ and patients with EGFR mutation usually benefit from targeted therapy. However, resistance to EGFR-targeted therapy inevitably occurs, with primary or acquired resistance mechanisms leading to treatment interruption and disease progression.

A meta-analysis of 15 studies speculated that tumor protein 53 (TP53) mutations are associated with primary resistance of EGFR-tyrosine kinase inhibitors (EGFR-TKIs) in NSCLC patients.^[3]^ TP53 mutation is the most common co-mutant gene in EGFR-mutant NSCLC, accounting for 55% to 65% cases.^[4]^ Several clinical studies have explored the role of TP53 mutations in influencing treatment response and drug resistance to EGFR-targeted therapy, which has proven to be an important factor in the poor prognosis of patients resistant to first-, second-, and third-generation EGFR-TKIs.^[5]^ Different subtypes of TP53 mutation may have different effects. Studies have shown that TP53 exon 8 mutant can identify a worse prognosis than exon 8 wild-type and other exon wild-type and mutant patient subgroups.^[6]^ However, an open, single-arm, prospective, multicenter phase II clinical trial conducted in China found that mutations in TP53 exons 6 and 7 were significantly associated with poorer progression-free survival (PFS) and overall survival (OS) than wild-type TP53.^[7]^

The present status of specific subtypes of TP53 mutation is not clear, and more studies and data are still needed to validate the findings. How to select the best treatment strategy for advanced NSCLC patients with EGFR-TP53 co-mutation should also be the focus of ongoing and future research.^[8]^ While both domestic and international scientists and clinicians have carried out in-depth investigations in this regard, no consensus has yet been reached. A retrospective study in China confirmed the advantages of combination chemotherapy with EGFR-TKIs compared with EGFR-TKIs monotherapy for the treatment of patients with EGFR-TP53 co-mutation,^[9]^ suggesting the importance of combination therapy. Another clinical trial (CTONG1706, apatinib combined with gefitinib versus gefitinib combined with placebo)^[10]^ showed that EGFR-TKIs combined with antiangiogenesis drugs could significantly improve the PFS of patients with EGFR-TP53 co-mutation compared with EGFR-TKIs alone. However, this trial had a small sample size and was not representative enough and hence needs validation via large sample studies. A real-world study of the efficacy of crizotinib combination therapy in overcoming mesenchymal-epithelial transition factor (MET) amplification-induced EGFR-TKIs resistance^[11]^ showed that in patients with TP53 mutations, patients receiving the combination therapy had longer OS than those receiving either crizotinib alone or chemotherapy alone, but this difference was not statistically significant. In addition, a real-world study of a large Chinese cohort showed that high PD-L1 expression was significantly associated with co-mutations in EGFR and tumor suppressor genes such as TP53, and patients with EGFR-TP53 co-mutations would likely benefit from anti-PD-1/PD-L1 therapy.^[12]^

Based on these studies, we speculated that TP53 mutations could affect the efficacy of EGFR-TKIs in patients with advanced NSCLC with EGFR mutation hence, it is paramount to explore effective treatment strategies. By comparing the treatment response and prognosis of TP53 mutation and TP53 wild type in patients with advanced NSCLC with EGFR mutation receiving EGFR-TKIs treatment, this study aims to clarify the influence of the EGFR-TP53 co-mutation on the efficacy of EGFR-TKIs, analyze effective treatment methods for EGFR-TP53 co-mutation, explore the effect of different subtypes of TP53 mutations on the efficacy of EGFR-TKIs, and guide clinical treatment.

## 2. Methods

### 2.1. Research object and data collection

#### 2.1.1. Study objective

In this retrospective analysis, we included patients with inoperable advanced NSCLC who were admitted to the respiratory department at the General Hospital of Northern Theater Command between January 2019 and December 2021 whose genetic test results showed EGFR mutation TP53 wild-type or EGFR-TP53 co-mutation and who received first-line treatment receiving EGFR-TKIs.

#### 2.1.2. Inclusion criteria

1. Patients with inoperable advanced NSCLC confirmed by imaging and histopathology;
2. The genetic test report was positive for EGFR mutation;
3. The first-line treatment offered was EGFR-TKIs;
4. Availability of imaging data, pathological reports, gene test reports, antitumor treatment history, and follow-up data.

#### 2.1.3. Exclusion criteria

1. Combined with co-mutants other than TP53 mutation;
2. Patients were treated with EGFR-TKIs after surgery;
3. No lesions were evaluated;
4. The Eastern Cooperative Oncology Group Performance Status (ECOG PS) scale was ≥4 points.

#### 2.1.4. Data collection

Patients were screened per the aforementioned criteria, and demographic information and clinical treatment data of the target population were collected. Data included sex, age, smoking history, ECOG-PS score, pathological type, clinical stage, genetic test date, gene mutation type (including TP53 mutation subtype), treatment history, drug efficacy, and clinical outcome follow-up information. Patients whose clinical outcomes were not collected in the hospital information system were collected by telephonic follow-up. Data collection ended on January 15, 2024. This study was reviewed by the Medical Ethics Committee of the General Hospital of Northern Theater Command.

### 2.2. Follow-up and efficacy evaluation

Efficacy was evaluated according to RECIST 1.1, and the objective response rate (ORR) was calculated. PFS is defined as the time from the beginning of treatment with EGFR-TKIs to the patient’s first progression. OS is defined as the time from the beginning of treatment with EGFR-TKIs to the end date of data collection or death, whichever came first.

### 2.3. Statistical analysis

SPSS 26.0 statistical software (IBM Corporation, Armonk) was used for all statistical analysis. Descriptive statistics were used to analyze demographic data and baseline characteristics. Mean and standard deviation were used to describe continuous variables that were normally distributed, and median and interquartile intervals were used to describe data that were non-normally distributed. The composition ratio was used to describe the counting data. Kaplan–Meier method was used to plot the survival curve and calculate the median overall survival (mOS) and median PFS (mPFS). T-test or Wilcoxon test were used to compare the mean values. Chi-square test or Fisher exact probability method was used to compare the ORR between groups. The survival rate between the groups was compared by the log-rank test. *P* <.05 was considered to indicate statistically significant differences.

## 3. Results

### 3.1. Age at first diagnosis, sex, smoking history, pathological type, and prognosis

From January 2019 to December 2021, 276 patients with NSCLC underwent genetic testing. Among these, 46 patients met the inclusion criteria: 20 patients with EGFR mutation only, and 26 patients with EGFR-TP53 co-mutation. Among the 46 patients included in this study, the mOS was 14.0 months for male patients and 28.0 months for female patients, with no significant difference between the sexes (*P* = .816). The median age of onset was 66 years (age range at first diagnosis: 42–80 years). Patients were categorized into 2 age groups: those aged ≥60 years and those aged <60 years. Among the 32 patients aged ≥60 years, 22 died, and among 14 patients aged <60 years, 8 died, with mOS of 28.0 months and 23.0 months, respectively. There was no significant difference in mOS among different age groups (*P* = .277). The mOS of 13 patients with smoking history was 27.0 months, and that of 33 patients without smoking history was 24.0 months (*P* = .778). The mOS of patients with squamous cell carcinoma was 13.5 months and that of patients with adenocarcinoma was 26.0 months. There was no significant difference in mOS between squamous cell carcinoma and adenocarcinoma (*P* = .105).

### 3.2. Comparison of the efficacy of TP53 mutant group and TP53 wild-type group on EGFR-TKIs

#### 3.2.1. Comparison of baseline demographic characteristics between the 2 groups

The included patients were divided into 2 groups according to whether TP53 was mutated, namely, the EGFR-TP53 co-mutation group (n = 26) and EGFR mutation TP53 wild-type group (n = 20).

The mean age of the co-mutation group was 63.04 ± 8.27 years, while that of the TP53 wild-type group was 65.70 ± 9.21 years. The mean age of the 2 groups was not statistically significant (*P* = .309).

In the co-mutation group, there were 13 male (50.0%) and 13 female (50.0%) patients. In the TP53 wild-type group, there were 7 male (35.0%) and 13 female (65.0%) patients. Chi-square test showed no significant difference in the male-to-female ratio between the 2 groups (*P* = .309).

The median ECOG-PS scores (lower quartile Q25, upper quartile Q75) of the co-mutation group and the TP53 wild-type group were 0.00 (0.00, 1.00) and 0.00 (0.00, 1.00), respectively. There was no significant difference in ECOG-PS scores between the 2 groups by rank sum test (*P* = .279).

In the co-mutation group, 10 (38.5%) patients had smoking history and 16 (61.5%) had no smoking history. In the TP53 wild-type group, 3 (15.0%) had smoking history and 17 (85.0%) had no smoking history. Chi-square test showed no significant difference in the proportion of smoking history between the 2 groups (*P* = .080).

In the co-mutation group, there were 2 (7.7%) cases of squamous cell carcinoma and 24 (92.3%) cases of adenocarcinoma. There were 20 (100.0%) cases of adenocarcinoma in the TP53 wild-type group. Chi-square test showed no significant difference in the proportion of pathological types between the 2 groups (*P* = .205).

#### 3.2.2. ORR comparison between the 2 groups

All patients in both groups were treated with EGFR-TKIs as first-line monotherapy. The ORR of the co-mutation group was 46.2%, compared to 75.0% in the TP53 wild-type group. The difference was statistically significant by chi-square testing (*P* = .049). The EGFR-TP53 co-mutation group had a worse response to EGFR-TKIs than the TP53 wild-type group (Table 1).

**Table 1:** ORR comparison between the 2 groups.

Group	CR + PR	SD + PD	*χ2*	*P*
EGFR-TP53 co-mutation	12 (46.2%)	14 (53.8%)	3.880	.049
EGFR mutation TP53 wild-type	15 (75.0%)	5 (25.0%)		

CR = complete remission, EGFR = epidermal growth factor receptor, ORR = objective response rate, PD = progressive disease, PR = partial remission, SD = stable disease, TP53 = tumor protein 53.

#### 3.2.3. Comparison of PFS between the 2 groups

**Figure 1.**
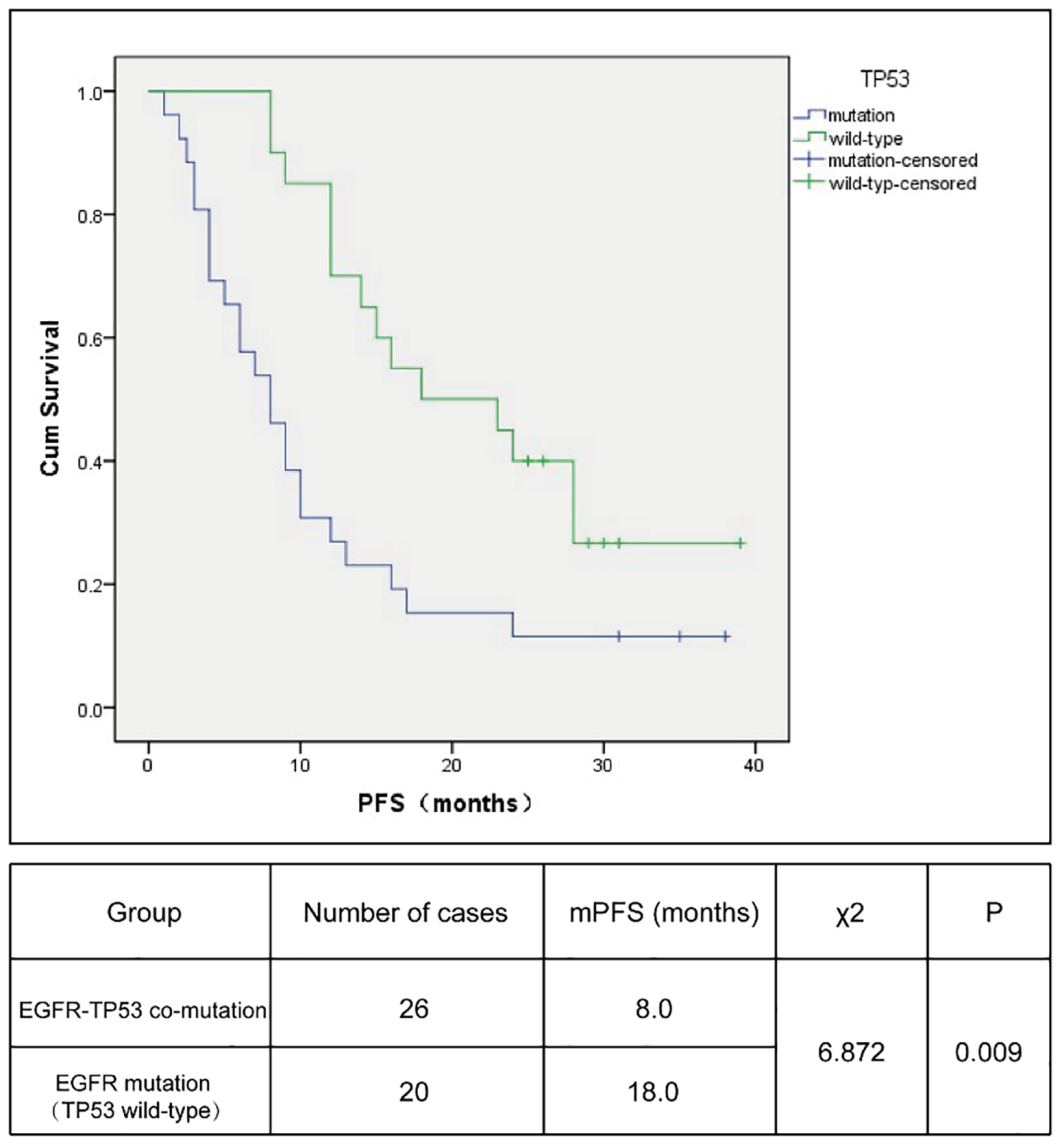
PFS curves of the co-mutation group and the EGFR-mutant TP53 wild-type group. The 46 included patients had a maximum PFS of 39.0 months and a minimum of 1.0 mo. In the co-mutation group, the longest PFS was 38.0 months, the shortest was 1.0 mo, and the mPFS was 8.0 mo. In the TP53 wild-type group with EGFR mutation, the longest PFS was 39.0 mo, the shortest was 8.0 months, and the mPFS was 18.0 mo. The mPFS of the 2 groups was statistically significant (*P* = .009). EGFR = epidermal growth factor receptor, mPFS = median progression-free survival, PFS = progression-free survival, TP53 = tumor protein 53.

As of January 15, 2024, the 46 included patients had a maximum PFS of 39.0 months and a minimum of 1.0 month. In the co-mutation group, the longest PFS was 38.0 months, the shortest was 1.0 month, and the mPFS was 8.0 months. In the TP53 wild-type group with EGFR mutation, the longest PFS was 39.0 months, the shortest was 8.0 months, and the mPFS was 18.0 months. The mPFS of the 2 groups was statistically significant (*P* = .009). The EGFR-TP53 co-mutation group had shorter mPFS after EGFR-TKIs treatment than the EGFR mutation TP53 wild-type group (Fig. 1).

A stratified analysis of the EGFR-TKIs type received as first-line treatment was performed in both groups. In the co-mutation group, 6 patients received first-generation EGFR-TKIs and 20 received third-generation EGFR-TKIs. In the TP53 wild-type group with EGFR mutation, 9 patients received the first-generation EGFR-TKIs, 1 received the second-generation EGFR-TKIs, and 10 received the third-generation EGFR-TKIs. The PFS (mean ± standard deviation) of the co-mutation group receiving first-generation TKIs was 5.17 ± 3.66 months, and the PFS of the EGFR-mutant TP53 wild-type group receiving first-generation TKIs was 15.50 ± 7.62 months (*P* = .011). The PFS of the co-mutation group receiving third-generation TKIs was 13.13 ± 11.12 months, and that of the EGFR-mutant TP53 wild-type group receiving third-generation TKIs was 24.60 ± 8.76 months (*P* = .009).

#### 3.2.4. mOS comparison between the 2 groups

**Figure 2.**
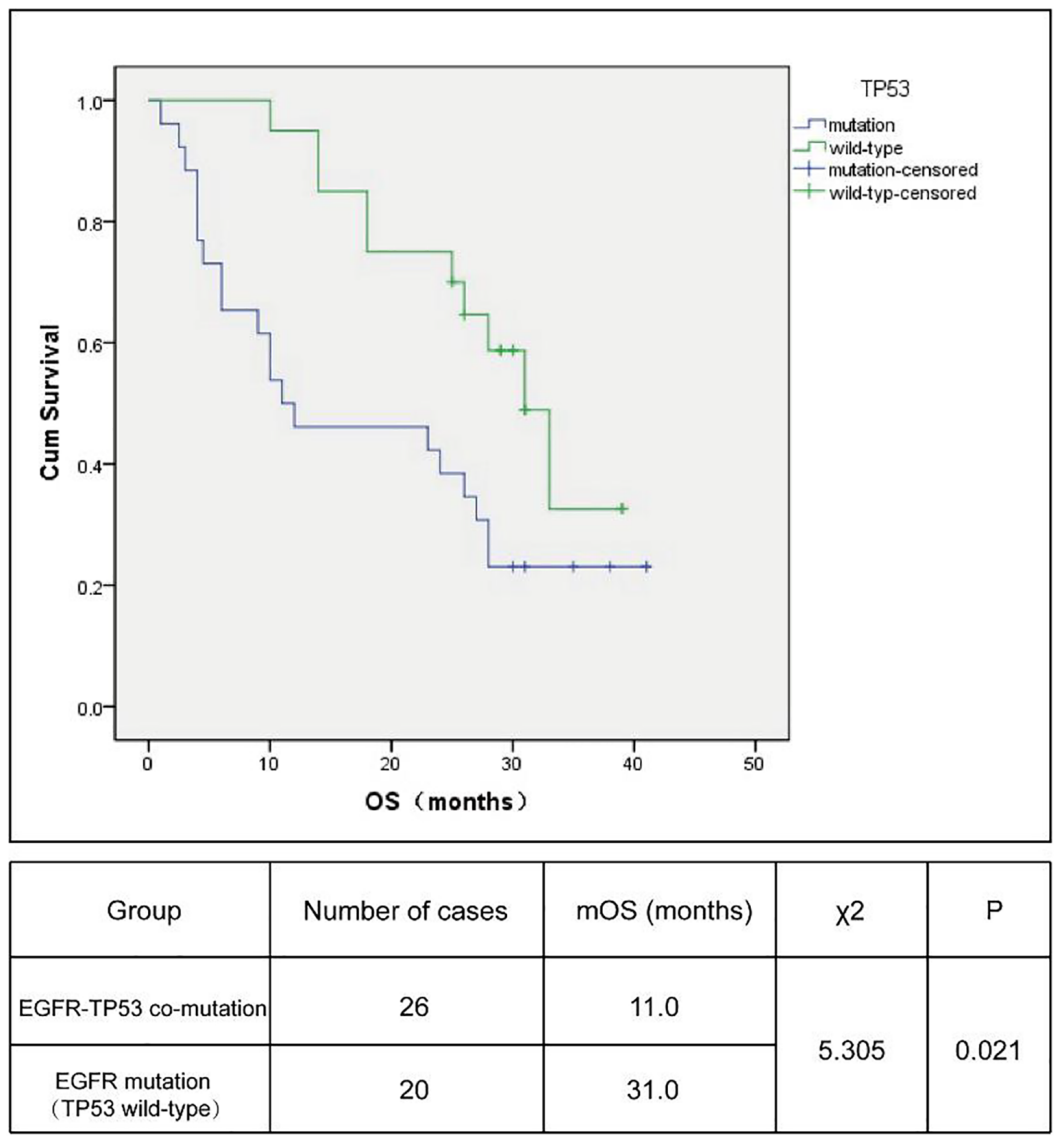
OS curves of the co-mutation group and the EGFR-mutant TP53 wild-type group. 30 of the 46 (65.2%) patients had died, and the longest OS was 41.0 mo and the shortest OS was 1.0 mo. Among the 26 patients in the co-mutation group, 20 (76.9%) died. The longest OS was 41.0 mo, the shortest was 1.0 mo, and the mOS was 11.0 mo. Ten of the 20 (50%) patients in the EGFR mutation TP53 wild-type group died; the longest OS was 39.0 mo, the shortest was 10.0 mo, and the mOS was 31.0 mo. The mOS of the 2 groups was tested by log-rank test (*P* = .021). EGFR = epidermal growth factor receptor, mOS = median overall survival, OS = overall survival, TP53 = tumor protein 53.

As of January 15, 2024, 30 of the 46 (65.2%) patients had died, and the longest OS was 41.0 months and the shortest OS was 1.0 month. Among the 26 patients in the co-mutation group, 20 (76.9%) died. The longest OS was 41.0 months, the shortest was 1.0 month, and the mOS was 11.0 months. Ten of the 20 (50%) patients in the EGFR mutation TP53 wild-type group died; the longest OS was 39.0 months, the shortest was 10.0 months, and the mOS was 31.0 months. The mOS of the 2 groups was tested by log-rank test (*P* = .021). The EGFR-TP53 co-mutation group had a shorter mOS than the EGFR mutation TP53 wild-type group (Fig. 2).

### 3.3. Comparison of the efficacy of EGFR-TP53 co-mutation group on the first-generation and the third-generation EGFR-TKIs

#### 3.3.1. mPFS comparison of the EGFR-TP53 co-mutation group that received gefitinib and osimertinib

The 26 patients in the EGFR-TP53 co-mutation group were divided into 2 groups. One group received first-generation EGFR-TKIs (all gefitinib, n = 6) as first-line therapy, and the other group received third-generation EGFR-TKIs (all osimertinib, n = 20) as first-line therapy.

As of January 15, 2024, the longest PFS of the gefitinib group was 12.0 months, the shortest was 2.0 months, and the mPFS was 3.0 months. The longest PFS of the osimertinib group was 38.0 months, the shortest was 1.0 month, and the mPFS was 9.0 months. There was a statistically significant difference in mPFS between the 2 groups (*P* = .022). Compared with gefitinib, first-line treatment with osimertinib significantly prolonged PFS (Table 2).

**Table 2:** mPFS comparison of the EGFR-TP53 co-mutation group that received gefitinib and osimertinib.

EGFR-TKIs	Number of cases	Number of progressive cases	mPFS (months)	*χ2*	*P*
Gefitinib	6	6	3.0	5.279	.022
Osimertinib	20	17	9.0		

EGFR = epidermal growth factor receptor, mPFS = median progression-free survival, TKIs = tyrosine kinase inhibitors, TP53 = tumor protein 53.

#### 3.3.2. mOS comparison of the EGFR-TP53 co-mutation group that received gefitinib and osimertinib

As of January 15, 2024, the longest OS of the gefitinib group was 24.0 months, the shortest was 3.0 months, and the mOS was 6.0 months. The longest OS of the osimertinib group was 41.0 months, the shortest was 1.0 month, and the mOS was 12.0 months. The mOS of the 2 groups was tested by the log-rank test (*P* = .041). Compared with gefitinib, first-line treatment with osimertinib significantly prolonged OS (Table 3).

**Table 3:** mOS comparison of the EGFR-TP53 co-mutation group that received gefitinib and osimertinib.

EGFR-TKIs	Number of cases	Number of deaths	mOS (months)	*χ2*	*P*
Gefitinib	6	6	6.0	4.195	.041
Osimertinib	20	14	12.0		

EGFR = epidermal growth factor receptor, mOS = median overall survival, TKIs = tyrosine kinase inhibitors, TP53 = tumor protein 53.

### 3.4. Prognosis analysis of second-line therapy in EGFR-TP53 co-mutation group

**Figure 3.**
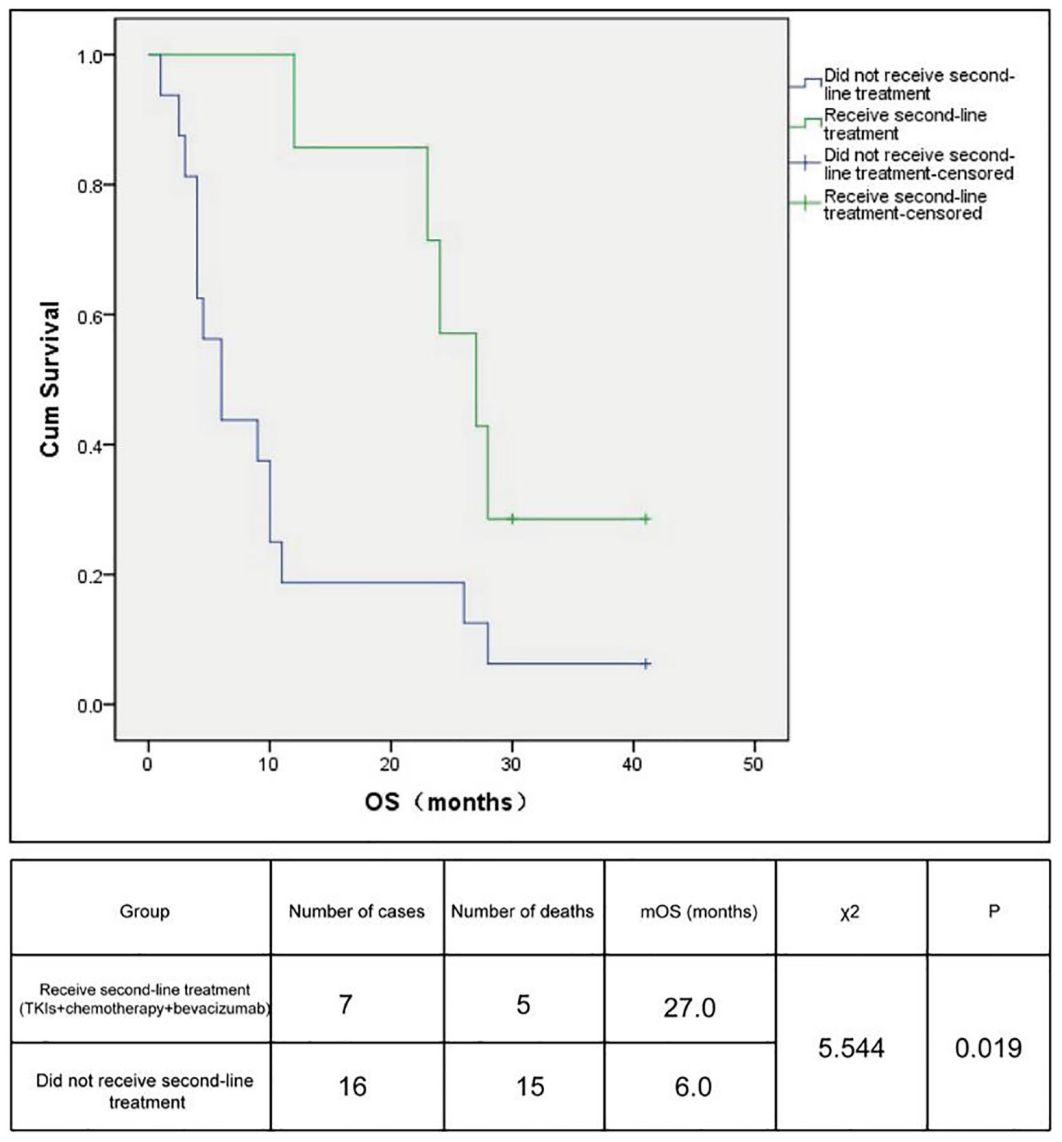
OS curves of EGFR-TP53 co-mutation receiving second-line therapy and not receiving second-line therapy after progression of first-line monotherapy TKIs. Of the 26 patients in the EGFR-TP53 co-mutation group, 23 had disease progression after first-line monotherapy EGFR-TKIs, of whom 7 had received second-line TKIs combined with platinum-containing double-drug chemotherapy (docetaxel or pemetrexed plus carboplatin or cisplatin) and bevacizumab. Sixteen patients did not receive second-line treatment and chose to continue with monotherapy TKIs or to abandon treatment. The longest OS was 41.0 months, the shortest was 12.0 months, and the mOS was 27.0 months in the 7 patients who received second-line treatment. Of the 16 patients who did not receive second-line treatment, the longest OS was 41.0 months, the shortest was 1.0 month, and the mOS was 6.0 months. The mOS of the 2 groups was tested by the log-rank test (*P* = .019). EGFR = epidermal growth factor receptor, mOS = median overall survival, OS = overall survival, TKIs = tyrosine kinase inhibitors, TP53 = tumor protein 53.

As of January 15, 2024, of the 26 patients in the EGFR-TP53 co-mutation group, 23 had disease progression after first-line monotherapy EGFR-TKIs, of whom 7 had received second-line TKIs combined with platinum-containing double-drug chemotherapy (docetaxel or pemetrexed plus carboplatin or cisplatin) and bevacizumab. Sixteen patients did not receive second-line treatment and chose to continue with monotherapy TKIs or to abandon treatment. The longest OS was 41.0 months, the shortest was 12.0 months, and the mOS was 27.0 months in the 7 patients who received second-line treatment. Of the 16 patients who did not receive second-line treatment, the longest OS was 41.0 months, the shortest was 1.0 month, and the mOS was 6.0 months. The mOS of the 2 groups was tested by the log-rank test (*P* = .019). The mOS of the group that received second-line combination therapy was significantly longer than the group that did not receive second-line treatment (Fig. 3).

### 3.5. Comparison of prognosis of different TP53 mutation subtypes in EGFR-TP53 co-mutant group

Among the 26 patients with EGFR-TP53 co-mutation, there were 4 different TP53 mutation subtypes: exon 5 (E5) mutation was observed in 8 cases and an mPFS of 5.0 months; exon 6 (E6) mutations were observed in 6 cases with an mPFS of 9.0 months; exon 7 (E7) mutation was observed in 5 cases with an mPFS of 8.0 months; and exon 8 (E8) mutation was observed in 7 cases with an mPFS of 6.0 months. There was no significant difference in mPFS among the different TP53 mutation subtypes (*P* = .232).

Among the 26 patients with EGFR-TP53 co-mutation, the mOS of the E5 mutant, E6 mutant, E7 mutant, and E8 mutant groups was 6.0 months, 24.0 months, 27.0 months, and 12.0 months, respectively. The difference in mOS among these groups was assessed using the log-rank test and found to be not statistically significant (*P* = .572).

## 4. Discussion

The development of targeted therapies has revolutionized treatment strategies for NSCLC with EGFR mutation, resulting in significant improvements in clinical outcomes. However, in most cases, TKIs inevitably develop resistance despite their good initial efficacy. The mechanisms of TKI resistance have not yet been fully elucidated and remain an open area of research.

The TP53 gene encodes the tumor suppressor protein p53 and is the most frequently mutated gene in all cancer types. TP53 mutations can induce carcinogenesis, tumor development, and resistance to treatment, affecting patient prognosis and response to treatment.^[13]^ In NSCLC, TP53 mutations are more common in squamous cell cancers than adenocarcinomas^[14]^ and are highly associated with smoking habits,^[15]^ but our study did not go further with these findings. One study^[16]^ found that co-mutation of TP53 was a predictor of TKIs efficacy and survival in NSCLC with EGFR mutation, independent of other parameters currently available. Furthermore, it may be an important factor in the risk stratification of NSCLC with newly diagnosed metastatic EGFR mutation. Previous literature has reported^[5]^ that among patients with advanced NSCLC who were EGFR-positive, those with TP53 co-mutation developed drug resistance earlier after first-line treatment with EGFR-TKIs. The same result was obtained in our study. Despite this, there are currently no effective drugs that can eliminate the carcinogenic function of TP53 mutations approved for clinical use. Therefore, it is of great significance to identify the best treatment plan and improve the prognosis of patients with advanced NSCLC with EGFR-TP53 co-mutation.

How to select EGFR-TKIs type in the single target treatment regimen for patients with co-mutation has not yet been reported in literatures or recommended by expert consensus. In actual clinical practice, some patients may be reluctant to accept chemotherapy or other combination therapy owing to chemotherapy intolerance or other reasons and hence choose single-agent targeted therapy. Combined with our study results, for these patients with advanced NSCLC with EGFR-TP53 co-mutation, single-agent osimertinib may be the preferred first-line treatment.

It is also worth exploring how to choose the second-line treatment for EGFR-TP53 co-mutation patients after the treatment of first-line monotherapy EGFR-TKIs progresses. Previous studies have shown^[17]^ that EGFR-TKIs targeted therapy combined with chemotherapy can increase the sensitivity of patients with EGFR-TP53 co-mutation advanced NSCLC to EGFR-TKIs targeted drugs and improve the overall prognosis of patients. Targeted synchronous chemotherapy can be used as the first-line treatment, and targeted combination chemotherapy can be selected after the development of single target therapy. Studies have also confirmed^[8]^ that wild-type p53 protein can inhibit new angiogenesis. TP53 mutation has an altered inhibitory effect on new angiogenesis, and TP53 mutation is associated with VEGF-A overexpression in NSCLC adenocarcinoma patients, suggesting potential therapeutic benefits of antiangiogenesis therapy in patients with EGFR-TP53 co-mutation. Two studies have shown that combination of antiangiogenic drugs with EGFR-TKIs significantly improves PFS in patients with EGFR-TP53 co-mutation compared to EGFR-TKIs alone.^[18,19]^ In our study, mOS was significantly prolonged in the group receiving second-line combination therapy (TKIs combined with chemotherapy and bevacizumab) compared to the group not receiving second-line therapy. These results suggest that multi-drug combination therapy, namely EGFR-TKIs combined with chemotherapy or EGFR-TKIs combined with antiangiogenesis or EGFR-TKIs combined with chemotherapy and antiangiogenesis therapy, may be a way to delay disease progression and prolong survival in cases where EGFR-TKIs monotherapy fails.

About 80% to 90% of TP53 mutations were concentrated in the DNA-binding domain region corresponding to the p53 protein in exons 5 to 8 of the gene,^[8]^ and all TP53 mutant regions in this study were in exons 5 to 8. Some studies^[6,20,21]^ have shown that in subtypes of TP53 mutations, the E8 mutation is associated with decreased disease control rate and significantly shortened mPFS and mOS. Analysis of data from a phase III randomized clinical trial (CTONG 0901)^[22]^ showed that mutations in exons 4 or 7 of TP53 were an independent adverse prognostic factor for patients with advanced EGFR-mutated NSCLC. In this study, there was no significant statistical difference in PFS and OS between different TP53 mutation subtypes in the EGFR-TP53 co-mutation group, which may be related to the small sample size of each group after stratification. Thus, further exploration of larger sample studies is still needed.

This study highlights the importance of considering TP53 mutations in the management of NSCLC with EGFR mutations. Our findings suggest that combination therapies may offer significant benefits, and further research is warranted to optimize treatment strategies for this patient population. However, this study has some limitations. First, it is a single-center retrospective study and a relatively small sample size. Second, this study did not explore the mechanism of EGFR-TKIs resistance, and the correlation between TP53 mutation and EGFR-TKIs primary resistance in NSCLC patients was only obtained from the literature. Moreover, TKIs combination therapy can not only combine chemotherapy and antiangiogenic therapy but also combine other TKIs, immunotherapy, and radiotherapy. Whether these combination therapies can benefit patients with EGFR-TP53 co-mutation is also worthy of further study.

## 5. Conclusions

TP53 mutations can significantly negatively affect the efficacy of EGFR-TKIs in patients with advanced NSCLC harboring EGFR mutation. Among patients with EGFR-TP53 co-mutation undergoing first-line EGFR-TKIs monotherapy, osimertinib was obviously superior to gefitinib. In the case of first-line EGFR-TKIs monotherapy progression, TKIs combined with chemotherapy and antiangiogenesis therapy could prolong the survival of patients. In TP53 exon 5 to 8 mutations, no mutation subtype was found to have a more conspicuous negative effect on the efficacy of EGFR-TKIs.

## Acknowledgments

We thank the Graduate School of China Medical University for its support and the General Hospital of the Northern Theater Command for providing data.

## Author contributions

**Writing – original draft:** Xiaohan Feng.

**Writing – review & editing:** Lei Liu.
